# Effect of Honey on Peridural Fibrosis Formation after Laminectomy in Rats: A Novel Experimental Study

**DOI:** 10.1155/2011/504967

**Published:** 2011-01-23

**Authors:** Majid Reza Farrokhi, Mohammad Vasei, Saeed Fareghbal, Atefeh Bakhtazad

**Affiliations:** ^1^Shiraz Neurosciences Research Center, Shiraz University of Medical Sciences, Shiraz, Iran; ^2^Shiraz Neurosciences Research Center, Chamran Hospital, Chamran Boulevard, 71966-93111 Shiraz, Iran; ^3^Department of Pathology, Shiraz Neurosciences Research Center, Shiraz University of Medical Sciences, 71966-93111 Shiraz, Iran

## Abstract

Despite progress in surgical techniques, some patients still face postoperative recurrence of pain. Recently, more attention has been focused on peridural fibrosis (PF), which may be responsible for recurrent pain after laminectomy or discectomy. Honey has been shown to exert anti-inflammatory effects on exposed tissues besides its well-known antibacterial properties. The aim of this study were to investigate the effects of honey on the prevention of postlaminectomy fibrosis formation in a rat model. A controlled blinded study was performed in 45 male adult white Sprague-Dawley rats that underwent laminectomy at the L5-L6 levels. They were divided into 3 groups (A, B, and C) of 15 rats each. Group A (sham) underwent laminectomy and group B was treated with normal saline at the laminectomy site. Rats in group C received 0.1 mL honey at the laminectomy site. All rats were killed 4 weeks after laminectomy. PF was found in 5 rats (33%) of control groups A and B, and in 2 rats (10%) in honey-treated laminectomy group C. The difference was not statistically significant. Wound healing was not affected, and there was no cerebrospinal fluid leakage. Although honey appears to be safe, it cannot cause a significant reduction of PF formation after lumbar laminectomy in rats.

## 1. Introduction

Each year, over one million people across the world undergo lumbosacral surgery following disc herniation, making it one of the most common treatments for spinal disorders [1, 2]. Despite progress in surgical techniques, some patients still suffer from postoperative recurrence of pain [3]. Failed back surgery syndrome (FBSS) refers to the presence of persistent and disabling pain in the hip, thigh, leg, or lower back in a patient who has undergone laminectomy with or without discectomy. Its incidence rate was reported at 5% to 30% [4]. Improper diagnosis and surgery, spinal stenosis, internal disc disruption, recurrent or retained disc, and peridural fibrosis (PF) cause FBSS [5–7]. Even if PF does not cause persistent pain, it can affect the outcomes of repeat operations done for recurrent herniation or stenosis, as it may lead to complications such as dural tearing, excessive bleeding and nerve injury. The success rate of repeated surgery for fibrosis is only from 30% to 35%, whereas 15% to 20% of patients report worsening of symptoms [3, 8, 9]. As a result, preventing fibrosis formation is preferable to trying to manage it. A number of agents have been used to prevent PF, including pharmaceutical gels, gelfoam, or free fat grafts [10]. Although they are used clinically as the most popular interpositional materials especially after laminectomy [10–12], their efficacy is not as impressive as their popularity [13, 14]. 

Honey has been principally used for its antibacterial effects since ancient times [15]. The main body of literature on medical use of honey includes its application over chronic wounds and burns as an antibacterial agent and a promoter of wound healing [16–18]. Some forms of medically-certified honeys have been licensed as a medical product for professional wound care in Europe and Australia [18, 19]. Different mechanisms of action have been proposed for honey. Its sugar content is so high that it hinders microbial growth [18, 20]. In addition, honey is hygroscopic, meaning that it can shrink the bacteria with the aid of its hyperosmolar properties [21–23]. 

In addition to its antibacterial properties, honey has anti-inflammatory effects. This anti-inflammatory action of honey reduces edema and an amount of exudates by down-regulating the inflammatory process. It also reduces pain by reducing both sensitization following inflammation-mediated prostaglandin synthesis and pressure on tissue resulting from edema [24]. 

Such a down-regulation of excessive inflammation may be a beneficial target to prevent peridural fibrosis in laminectomized subjects. The aim of this preclinical study was to determine whether honey had a similar effect on PF in a rat postlaminectomy model.

## 2. Materials and Methods

### 2.1. Animals in the Study

Forty-five male adult white Sprague-Dawley rats weighing averagely 275 g were used for this study after approval was obtained from the University Committee on Animal Resources. The study was performed at the Laboratory Animals Center of Shiraz University of Medical Sciences. Sample size was calculated based on a 90% power, and a type I error rate of 5%, which necessitated at least 15 cases in each group.

### 2.2. Surgical Procedure

Intravenous anesthesia was induced with ketamine (35 mg/kg) and xylazine (10 mg/kg). The rats were placed in a prone position and the surgical field was prepared with povidone-iodine soap and solution. The area was draped in an aseptic manner. A midline skin incision was made from L2 to S1, followed by bilateral paramedian fascial incisions through the muscle, and carried down to the level of the spinal laminae under magnification with an operative microscope (880, Carl Zeiss Inc., Jena, Germany). The spine processes of the L5 and L6 vertebrae were removed and fine neurosurgical punches were used for total laminectomies at the L5 and L6 levels in each rat. The laminectomy defects measured approximately 5 × 5 mm. Then the ligamentum flavum and epidural fat were removed and the dura mater was exposed. 

The rats were randomly divided into 3 groups of 15 rats each, utilizing simple randomization. Every rat received a random computer-generated number, which corresponded to one of the three groups. In group A, the sham-operated group, only laminectomy was performed and no treatment was used. In group B, the second control group, 0.1 mL normal saline was applied to the peridural space after laminectomy. In treatment group C, honey prepared from beehives in pastures of the Dena mountain range was applied to the peridural space. 100 g honey was dissolved in 10 mL distilled water. Honey was sterilized by 0.22-*μ*m filtration (Millipore, MA, USA). 0.1 mL sterile honey was used at the laminectomy site. The same preparation of honey was used for all cases to solve the issue of standardization. The wound was closed in layers.

No other medical treatment that could influence the potential effects of honey was used. The rats were housed separately during the first two postoperative days. Their neurological status was followed closely, and postoperative recovery was good in all animals with no neurological deficits.

All rats were followed up for 4 weeks and were then killed by pentobarbital (150 mg/kg) on postoperative day 28.

### 2.3. Preparation of Specimens

Lumbar specimens were removed en bloc and fixed in 10% buffered formalin. After 72 h, all specimens were cut down to the laminectomy site with a circular saw to provide complete visualization of possible fibrosis formation.

### 2.4. Macroscopic Exam

All specimens were grossly evaluated by a blinded observer who assessed the whole anatomy of fibrosis formation according to a rating system previously described by Einhaus et al. [25]. This rating system was used to assess the amount and tenacity of the fibrosis formation (Tables 1 and 2).

### 2.5. Microscopic Exam

The specimens were decalcified and stained with hematoxylin and eosin (H&E) and elastica-van Gieson stain, and evaluated to measure the extent of fibrosis formation (Table 3). A neuropathologist who was unaware of which group the animals had come from evaluated all specimens.

### 2.6. Statistical Analysis

SPSS software version 14 (SPSS, Inc., Chicago, IL, USA) was used for the statistical analysis. The primary outcome variable was considered to be the presence or absence of fibrosis. We also calculated three scores for each rat based on amount, tenacity, and extent, in addition to the sum of the scores. The data were compared between groups with the Kruskal-Wallis and Chi-square tests. Poisson regression, as performed by SAS (version 9.1), was used to calculate relative risks. *P* < .05 was considered statistically significant.

## 3. Results

There was no wound infection or skin inflammation in any of the rats treated with honey. All 45 rats (15 in each group) were active, ambulatory, and healthy when they were killed.

### 3.1. Gross Examination and Tenacity

Rating system described by Einhaus et al. [25] suggests scores 0 to 3 to assess the amount and tenacity of fibrosis formation (Tables 1 and 2) and measure histological changes and the extent of fibrosis formation (Table 3). Macroscopic exam scores of all rats which had fibrosis are presented in Table 4. There were no statistically significant differences between groups. Of the 3 study groups, the two control groups had the highest numbers of rats with fibrosis. Groups A and B each had 5 rats with fibrosis in different amounts and tenacity (Tables 5 and 6). In group C, which had received honey, PF was identified in 2 rats with a total score of 7 (Table 7). The number of rats that had fibrosis was 5 in the sham, 5 in the normal saline, and 2 in the honey group. Using Poisson regression, the relative risk for fibrosis formation for saline versus sham was calculated to be 1.0 (confidence interval = 0.29 to 3.5), and the relative risk for honey versus sham was 0.40 (confidence interval = 0.08 to 2.1); *P* = .27.

The total scores were calculated by adding the gross score, tenacity score, and histology score for each rat with fibrosis in each of the 3 groups (Tables 5–7), and the sum of the scores is given in the total score column. Table 4 also gives the total scores for all 3 groups. 

### 3.2. Microscopic Evaluation

**Figure 1:**
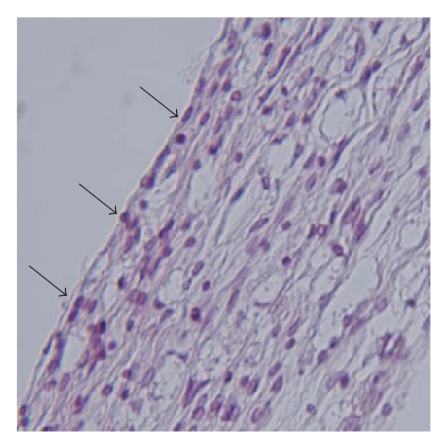
Peridural fibrosis at the sham operated (untreated; group A) site showing increased numbers of fibroblasts which are consistent with fibrosis formation (Arrows; Hematoxylin and eosin; ×400).

**Figure 2:**
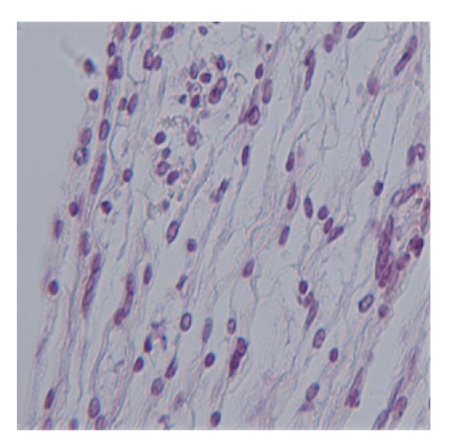
Laminectomy site treated with honey. Note mild infiltration of chronic inflammatory cells, few fibroblasts, and hemosiderin-bearing macrophages, which are consistent with a regular healing response (Hematoxylin and eosin; ×400).

**Figure 3:**
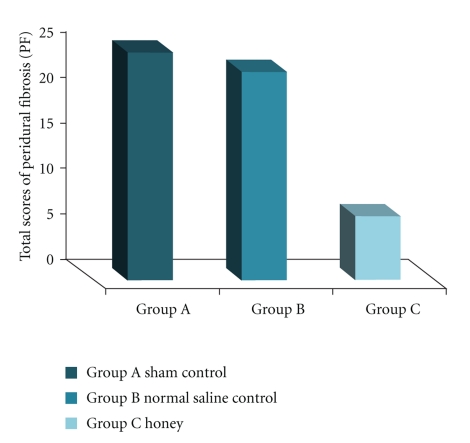
Comparison of PF* (Total scores) in the three groups (*P* = 0.287). *Peridural Fibrosis.

Histologic assessment of 5 rats with PF in groups A and B (Figure 1) showed hypervascularized granulation tissue, fibroblasts, and chronic inflammatory infiltrates that filled the laminectomy space and were in direct contact with the dura. Thick fibrosis formation with numerous enlarged sinusoidal vessels completely adhered to the dura was observed in 2 rats in group A. Only moderate chronic inflammation characterized by fibroblasts infiltration and hypervascularized granulation tissue was found in 2 rats in group C (Figure 2) with no evidence of fibrosis formation. The total scores for macroscopic and microscopic evaluations in all 3 groups were compared (Figure 3). There was no significant difference between the total scores in each group (*P* = .287).

## 4. Discussion

This study included 45 rats in 3 groups of 15 rats each to compare the ability of honey to prevent PF in sham, normal saline-treated, and honey-treated animals. Although the results suggested that animals treated with honey had less fibrosis (in figures) than sham or normal saline-treated animals after laminectomy, such an effect was not shown to be significant statistically. 

Honey has been used since ancient times. Ancient Egyptians, Assyrians, Chinese, Greeks, and Romans all used honey to treat gut wounds [15]. Avicenna, a great Iranian scholar, believed that honey was effective in local treatment of wound infections [26]. It is worthy to mention that even many religious quotes including the holy Qur'aan noted the curative properties of honey 1,400 years ago [27].

Honey contains various vitamins, minerals, and amino acids as well as glucose and fructose and is popular as a natural food [28]. It is used not only as a natural food but also as a traditional medicine for health care, in beauty products and anti-inflammatory skin care. Other aspects of its use indicate that it also has functions such as antibacterial, antioxidant, antitumor, anti-inflammatory, antibrowning, and antiviral [29]. 

The principal application of honey in medicine covers its antibacterial and wound-protecting activity, which makes it useful in the protection of burns and chronic wounds. The antimicrobial activity has been reported to be due to its high osmolarity, acidity, and the presence of hydrogen peroxide and unidentified substances from floral sources [28, 29]. 

In addition to its antibacterial properties, honey has anti-inflammatory effects which have been extensively observed clinically [30–32]. The anti-inflammatory action of honey reduces edema and the amount of exudates by down-regulating the inflammatory process [16]. Some studies have shown that honey works through modulating the inflammatory response and controlling angiogenesis and proliferation of fibroblasts and epithelial cells. Tonks et al. [31, 32] discovered that a 5.8-kDa component of manuka honey stimulates the production of TNF-*α* in macrophages via Toll-like receptor 4 [33]. Ahmad et al. [34] revealed the suppressive activity of honey towards thrombin-induced reactive oxygen species (ROS) production by phagocytes which could be beneficial in the interruption of some pathological progress. In another study, van den Berg et al. [35] showed that buckwheat honey was most effective in reducing ROS levels. They concluded that the major antioxidant properties in buckwheat honey derive from its phenolic constituents, which are present in relatively large amounts. Its phenolic compounds may also be responsible for antibacterial activity, whereas its low pH and high free acid content may assist wound healing. All these studies reveal the anti-inflammatory and immune-modulatory effects of honey, as well as some modifications in the action of certain cytokines and cells (mostly fibroblasts). This was the rationale over which we decided to evaluate the effect of honey on reducing PF at the laminectomy site. 

In light of the scores we obtained for gross and histological features, although honey caused less fibrosis both quantitatively and qualitatively than the two control groups, the differences were not statistically significant, possibly because of the small sample size. We are aware of no other experimental studies in rats that were designed to compare the control and treatment groups used here. Conditions in the animal laboratory are standardized and 15 rats were studied for each group—a reasonable number to detect treatment effects without having to kill more animals than necessary. However, because we compared the existence (1) versus the nonexistence of fibrosis (0), this limited the conclusions that can be drawn from our statistical analysis. This study may best cast as a pilot study so that in future, more highly powered studies with larger numbers of animals be carried out. 

The benefit of performing an animal experiment, including our study, resides in its ability to eliminate placebo effects. Many human trials suffer from such effect when certain individual beliefs or suggestions may influence their response to treatment [36]. There is modest evidence that the application of honey to the laminectomy wound is safe and causes no complications. The reduction we found in the total score for PF from 25 in the sham-operated group to 7 in the honey-treated group may justify a pilot study in humans. Treatment with honey after laminectomy may decrease pain via its anti-inflammatory effects [37, 38] in addition to its ability to reduce PF.

## 5. Conclusions

The prevention of PF after laminectomy is a major challenge in spine surgery. This study sheds new light on the subject and suggests that more detailed histological studies in other mammals or even humans may be warranted to further investigate the ability of honey to prevent fibrosis and pain. Rigorous controlled trials should be undertaken in groups of patients who undergo lumbar surgery with and without the application of honey to help determine the ultimate value of honey in the prevention of PF.

## Figures and Tables

**Table 1:** Scoring system to record gross anatomical changes.

Score	Amount of fibrosis
0	None
1	Partial fibrosis of the dura at the laminectomy site
2	Total fibrosis of the dura at the laminectomy site
3	Fibrosis extended beyond dura thickness

**Table 2:** Qualitative scoring system to assess fibrosis formation.

Score	Tenacity of fibrosis
0	No observed scar tissue
1	Scar tissue, separation from dura requires no dissection
2	Separation from dura requires blunt dissection
3	Separation from dura requires sharp dissection

**Table 3:** Scoring system to assess histological changes.

Score	Histology
0	No scar tissue
1	Moderate chronic inflammation
2	Severe chronic inflammation
3	Thick fibrosis formation

**Table 4:** Scores in all 3 groups for macroscopic changes indicating fibrosis formation.

Groups (*n* = 15)	Gross (amount) score			Tenacity score			Total score

	1	2	3	1	2	3	
Sham	2	2	1	2	3	0	17
Normal saline	2	3	0	1	4	0	17
Honey	2	0	0	1	1	0	5

**Table 5:** Results and scores in 5 animals with fibrosis in group A (sham).

Specimen	Slide number	Gross (amount) score	Tenacity Score	Histology score	Total scores
1	4	2	2	2	6
2	7	3	2	3	8
3	10	2	1	1	4
4	12	1	1	1	3
5	14	1	2	1	4

					25

**Table 6:** Results and scores in 5 animals with fibrosis in group B (normal saline).

Specimen	Slide number	Gross (amount) score	Tenacity score	Histology score	Total scores
1	1	1	2	1	4
2	3	2	2	1	5
3	7	2	2	2	6
4	9	2	1	1	4
5	13	1	2	1	4

					23

**Table 7:** Results and scores in 2 animals with fibrosis in group C (Honey).

Specimen	Slide number	Gross (amount) score	Tenacity score	Histology score	Total scores
1	4	1	1	1	3
2	11	1	2	1	4

					7
